# Overexpression of NLRP12 enhances macrophage immune response and alleviates herpes simplex keratitis

**DOI:** 10.3389/fcimb.2024.1416105

**Published:** 2024-07-25

**Authors:** Jiaxuan Jiang, Di Zhang, Wei Liu, Jingya Yang, Fan Yang, Junpeng Liu, Kai Hu

**Affiliations:** ^1^ Department of Ophthalmology, Nanjing Drum Tower Hospital, Affiliated Hospital of Medical School, Nanjing University, Nanjing, China; ^2^ Department of Ophthalmology, Linyi Bright Eye Hospital, Linyi, China

**Keywords:** herpes simplex keratitis, herpes simplex virus type 1, NLRP12, macrophage, antiviral, pyroptosis

## Abstract

**Introduction:**

Herpes simplex keratitis (HSK) is a blinding disease caused by corneal infection of Herpes simplex virus type 1 (HSV-1). Effective clearance of HSV-1 from the infected cornea is crucial for HSK management. Macrophages play an important part in the innate immune defense against viral infections. This study investigates the immunomodulatory role of NLRP12 in macrophage immune response during HSV-1 infection.

**Methods:**

NLRP12 expression post-infection was assessed in various macrophage cell lines. Overexpression of NLRP12 was achieved by lentiviral transfection, and its effect on HSV-1 replication and immune responses were examined. Mechanistic insights into the role of NLRP12 were explored using immunofluorescence and Western Blot. For *in vivo* studies, ocular adoptive transfer of NLRP12-overexpressing bone marrow derived macrophages (BMDMs) was performed. HSV-1 viral loads, HSK symptoms, and macrophage-mediated immune responses were investigated.

**Results:**

A significant decrease in NLRP12 expression post-infection was observed in various macrophage cell lines. Overexpression of NLRP12 in macrophages reduced HSV-1 replication. Mechanistically, overexpression of NLRP12 triggered early and robust pyroptosis in response to HSV-1 infection, inducing interleukin (IL)-18 production and activating downstream antiviral responses through the JAK-STAT signaling pathway. In vivo, ocular adoptive transfer of NLRP12-overexpressing BMDMs to mouse corneas alleviated HSK damage and reduced HSV-1 viral loads. NLRP12-overexpressing BMDMs improved antiviral responses in the cornea and promoted the maturation of corneal-infiltrating macrophages and dendritic cells. Additionally, NLRP12-overexpressing BMDMs amplified the adaptive immune response in the submandibular draining lymph nodes.

**Discussion:**

These findings highlight the role of NLRP12 in macrophage-mediated immune response against HSV-1 infection and suggest its potential for possible immunotherapy for HSK.

## 1 Introduction

Herpes simplex keratitis (HSK), caused by corneal infection with herpes simplex virus type 1 (HSV-1), is a leading cause of infectious blindness worldwide (Hazlett et al., 2023). The replication of the virus within the corneal tissue and its subsequent transmission lead to severe tissue damage and pathological injury. Therefore, rapid and effective clearance of the virus is crucial for HSK management (Koganti et al., 2021). Current therapeutic strategies of antiviral pharmacotherapy have shown limited success (Hoffman, 2020), highlighting a demand for the development of immunomodulatory targets for the clearance of HSV-1 and the treatment of HSK.

The pathogenesis of HSK involves complex interactions between the virus and the host’s immune defenses. The innate immune response to HSV-1 is particularly critical in containing the infection (Zhu and Viejo-Borbolla, 2021). Macrophages play an important role in the innate immune system and serve as one of the first leukocytes to infiltrate the corneas following HSV-1 infection (Lobo et al., 2019). Upon recognition of HSV-1, macrophages phagocytose infected cells or viral particles for degradation. After phagocytosis, macrophages can also present processed viral antigens on the surface via Major Histocompatibility Complex class II (MHC II) molecules, which is essential for the activation of CD4^+^ T cells, CD8^+^ T cells and other immune effectors in the draining lymph nodes (dLNs). Additionally, activated macrophages secrete a number of cytokines and chemokines, notably interferons (IFNs), to regulate immune cells and amplify the immune response (Koujah et al., 2019). Following the containment of the virus, macrophages also play an essential role in restoring homeostasis in the cornea and facilitating tissue repair (Mu et al., 2021). Research has demonstrated that the depletion of ocular macrophage populations via dichloromethylene diphosphonate exacerbates HSK lesions (Mott et al., 2007), indicating the therapeutic potential of bolstering macrophage-mediated immunity in HSK intervention.

Nucleotide-binding oligomerization domain (NOD)-like receptors (NLRs) are a family of cytoplasmic pattern recognition receptors that are mainly expressed within immune cells, particularly those of myeloid lineage such as macrophages, dendritic cells, and neutrophils (Babamale and Chen, 2021). NLRs play a crucial role in sensing intracellular pathogens and danger signals, triggering a specialized form of cell death known as pyroptosis. Activation of NLRs leads to oligomerization and recruitment of the adaptor protein apoptosis-associated speck-like protein containing a CARD (ASC), leading to the formation of the inflammasome complex. The inflammasome then activates caspase-1 (CASP1), which in turn cleaves interleukin-1β (IL-1β) and interleukin-18 (IL-18) into their mature forms and also cleaves gasdermin D (GSDMD) for membrane pore formation and the release of intracellular contents (Rao et al., 2022; Huston et al., 2023). The NLR signaling pathway and the resultant pyroptosis represent important mechanisms by which the innate immune system responds to intracellular infections.

NLRs are involved in antiviral immune response through inflammasome assembly that recognizes viral components or cellular stress signals induced by viral infections (Babamale and Chen, 2021). In the context of HSV-1 infection, in THP-1 cells the immune response against the virus is dependent on NLR family pyrin domain containing 3 (NLRP3), ASC, and CASP1 (Karaba et al., 2020). In murine microglia, activation of the NLRP3 inflammasome by HSV-1 triggers GSDMD-dependent pyroptosis, leading to the generation of active CASP1 and the release of mature IL-1β (Hu et al., 2022). Furthermore, the stimulator of interferon genes (STING) has been shown to interact with NLRP3 to promote inflammasome activation for the host defense against HSV-1 (Wang et al., 2020). Research by Coulon et al. has revealed that virulent HSV-1 strains induced early expression of NLRP3, NLRP12 and IFI16 inflammasomes (Coulon et al., 2019). While NLRP3 is the most studied NLR concerning viral infections (Zhao and Zhao, 2020), the roles of other NLR family members, such as NLRP12, in the immune response to HSV-1 are less defined and warrant further investigation.

The NLR family pyrin domain containing 12 (NLRP12) is a member of the NLRs family, and plays a key role in modulating inflammation and innate immune response (Tuncer et al., 2014). Beyond its regulatory role in the production of pro-inflammatory cytokines, studies have also highlighted the potential of NLRP12 to inhibit pathogen replication within host cells via pyroptosis (Tuladhar and Kanneganti, 2020). As an inflammasome sensor, NLRP12 initiates pyroptosis in response to infections by *Yersinia pestis* (Vladimer et al., 2012), *Plasmodium chabaudi* (Ataide et al., 2014), and *Toxoplasma gondii* (Znalesniak et al., 2017; Rajabi et al., 2022), which results in the secretion of IL-1β and IL-18 and a reduction in the pathogen burden. As mentioned above, Coulon et al. have reported that virulent HSV-1 strains triggered early induction of NLRP3, NLRP12, and IFI16 inflammasomes, which were associated with inflammatory stromal keratitis (Coulon et al., 2019). However, the specific role of NLRP12 in the antiviral immune responses beyond the inflammatory processes, remains to be fully clarified.

Given the importance of macrophages in the frontline defense against HSV-1 and the emerging significance of NLRP12 in immune regulation, our study aimed to explore the role of NLRP12 in macrophage-mediated antiviral defense during HSV-1 infection and its implications for HSK. We first observed a significant downregulation of NLRP12 across different macrophage cell lines post-infection. Overexpression of NLRP12 in RAW264.7 macrophages significantly decreased HSV-1 levels. Mechanistically, NLRP12 overexpression induced early and robust cell pyroptosis, leading to increased IL-18 production and activating the JAK-STAT signaling pathway. *In vivo*, adoptive transfer of NLRP12-overexpressing macrophages to HSK mice reduced HSV-1 viral load and ameliorated the clinical symptoms of HSK, promoting a stronger immune response within the cornea and draining lymph nodes. Our research offers novel insights into the management of HSK and other viral infectious diseases.

## 2 Materials and methods

### 2.1 RNA Sequencing Analysis

RNA-sequencing analysis was performed by Annoroad Gene Tech. (Beijing) Co., Ltd. Total RNA was isolated from mock-infected and HSV-1-infected BMDMs using TRIzol reagent (T9108, Takara, Japan). The quality and quantity of RNA were evaluated using the Agilent 2100 Bioanalyzer with the RNA Nano 6000 Assay Kit (Agilent Technologies, California, USA). Poly-A-tailed RNA libraries were constructed using MGIEasy RNA Library Prep Kit V3.0 and sequenced on the DNBSEQ-T7 platform.

### 2.2 Cell culture, infection, and inhibition treatment

Bone marrow-derived macrophages (BMDMs) were isolated according to previously established protocols (Mendoza et al., 2022) and characterized as shown in **Supplementary Figure S1**. BMDMs, mouse monocyte-macrophage (RAW264.7) cells (TCM-C766, Haixing Biosciences, China), and Vero cells (TCO-C022, Haixing Biosciences, China) were cultured in Dulbecco’s modified Eagle medium (DMEM, 11965092, Gibco, USA). Human monocytic (THP-1) cells (ZQ0086, Shanghai Zhong Qiao Xin Zhou Biotechnology, China) were cultured in RPMI 1640 medium (11875093, Gibco, USA). All complete medium were supplemented with 10% fetal bovine serum (FBS, 10091148, Gibco, USA) and 1mM penicillin-streptomycin (15140122, Gibco, USA). THP-1 cells were differentiated into macrophages with 100ng/ml Phorbol 12-myristate 13-acetate (PMA, TQ0198, TargetMol, China). HSV-1 strain McKrae was used for infection at a multiplicity of infection (MOI) of 1. RAW264.7 cells were pre-treated with 20 µM VX-765 (T6090, TargetMol, China) to inhibit pyroptosis and with 50 ng/ml IL-18 binding protein (IL-18BP) (HY-P75841, MCE, China) to inhibit IL-18 activity.

### 2.3 Lentiviral transfection

Lentiviral vectors were produced by GENECHEM Biotechnology (Shanghai, China). RAW264.7 cells or BMDMs were seeded at a density suitable for reaching 50–70% confluence at the time of transfection. The next day, Control-Lentivirus (LV-Ctrl) or Lentivirus expressing NLRP12 (LV-NLRP12) was added (MOI=30 for RAW264.7 cells, MOI=30 for BMDMs) in the presence of HistransG A (REVG004, GENECHEM Biotechnology, China) to enhance transduction efficiency. After 16 hours, the medium was replaced with fresh complete growth medium.

### 2.4 Animal model and assessment

The Animal Research Ethics Committee of Nanjing Drum Tower Hospital granted approval all animal experiments, adhering to the guidelines of the Vision and Ophthalmology Research Society. Female C57BL/6 mice, 6–8 weeks old, were purchased from the Animal Center of Yangzhou University and housed in the Animal Experimental Center of Nanjing Drum Tower Hospital (Nanjing, China) under controlled conditions (25°C, 40–60% humidity). For adoptive transfer, mice received subconjunctival injections of BMDMs transfected with control or NLRP12-expressing lentivirus (10^4^ cells/eye) as depicted in **Supplementary Figure S2A**. One day later, corneal scratching and HSV-1 strain McKrae (1 × 10^6^ PFU/ml) inoculation were performed as previously described (He et al., 2022; Wang et al., 2022; Shen et al., 2024) (**Supplementary Figure S2B**).

The grading of keratitis severity was conducted using a 0–4 scale HSK score: 0 = normal, no epithelial or punctate lesions and no edema or stromal opacity; 1 = stellate epithelial lesions or mild edema and stromal opacity, the iris is visible; 2 = dendritic or atlas-like epithelial lesions occupying <25% of the cornea, or stromal edema, cloudy lesions less than half the diameter of the cornea, the iris is visible; 3 = epithelial dendritic or atlas-like lesions occupying 25% to 50% of the cornea, or stromal edema, cloudy lesions greater than half the diameter of the cornea, iris partially invisible; 4 = epithelial dendritic or atlas-like lesions occupying >50% of the cornea, or severe stromal edema and opacity with completely invisible iris.

For assessing corneal fluorescein score (CFS), a small amount of 1% fluorescein sodium dye (Jingming, Tianjin, China) was applied to the inferior fornix of the mice’s conjunctiva. The corneal epithelial lesions, when observed under cobalt blue light, appeared yellow-green. 0–3 points were graded for each of the divided four regions of the cornea according to positive staining area. The final CFS score is the sum of the scores from the four regions (He et al., 2022; Wang et al., 2022; Shen et al., 2024).

### 2.5 Plaque assays and 50% tissue culture infectious dose (TCID50) assay

Tear samples were collected using eye swabs and stored in DMEM. Cell culture supernatant samples were collected at specified time points. For plaque assay, Vero cells were seeded into 12-well plates and infected with serial dilutions of the samples. The monolayers were overlaid with agarose, and after 48–72h, fixed with 4% paraformaldehyde and stained with crystal violet. For TCID50 assay, Vero cells were seeded in 96-well plates. After 24 hours, the supernatants were replaced with DMEM containing 2% FBS. Vero cells were then infected with 10-fold serial dilutions of the samples for 48–72h. The cytopathic effects in each dilution gradient were counted under a light microscope to calculate TCID50 by the Reed-Muench method (Capelli et al., 2020).

### 2.6 Western blot analysis

Cells were lysed using RIPA buffer (R0010, Solarbio, Beijing, China), and proteins were separated by SDS-PAGE and transferred to PVDF membranes (IPVH0010, Millipore, USA). HSV-gB and HSV-gD proteins were separated on 8% gels. NLRP12, CASP1, N-GSDMD, STAT1, STAT1 (Phospho-S727), STAT4, Phospho-STAT4 (Tyr693) and β-actin proteins were separated on 10% gels. Clv-CASP1, IL-18 and IL-1β proteins were separated on 12.5% gels. The membranes were then blocked with Y-Tec 5min Rapid Blocking Buffer (YWB0501, Yoche, China) and incubated overnight with primary antibodies against NLRP12 (1:1000, YT7568, Immunoway, USA), HSV-gB (1:500, sc-56987, Santa Cruz, USA), HSV-gD (1:500, sc-21719, Santa Cruz, USA), CASP1 (1:2000, 81482–1-RR, Proteintech, China), N-GSDMD (1:2000, ab215203, Abcam, UK), clv-CASP1 (1:500, AY0406, Abways, China), IL-18 (1:500, A1115, ABclonal, China), IL-1β (1:1000, ab254360, Abcam, UK), STAT1 (1:1000, CY5227, Abways, China), STAT1 (Phospho-S727) (1:1000, BM4541, Boster, USA), STAT4 (1:1000, BA0622–2, Boster, USA), Phospho-STAT4 (Tyr693) (1:1000, CY6503, Abways, China), and β-actin (1:20000, 81115–1-RR, Proteintech, China). After incubating with HRP-conjugated secondary antibodies (SA00001–1, SA00001–2, Proteintech, China), signals were detected using an ECL kit (BMU102-CN, Abbkine, China). Band density was quantified with ImageJ software.

### 2.7 RNA extraction and real-time quantitative PCR (RT-qPCR)

Total RNA was extracted using FreeZol Reagent (R711, Vazyme Biotech Co., Ltd., Nanjing, China). cDNA was synthesized using HiFiScript gDNA Removal RT MasterMix (CW2020, CWBIO, China). RT-qPCR was performed using MagicSYBR Mixture (CW3008, CWBIO, China) on Applied Biosystems QuantStudio 5 Real-Time PCR System (Thermo Fisher Scientific, USA). Gene expression was normalized to β-actin and analyzed by the 2^−ΔΔCT^ method. Primer sequences are listed in **Table 1**.

**Table 1:** Primers for RT-qPCR.

Gene	Forward Primer	Reverse Primer
TNF-α	TGATGACATCAAGAAGGTGGTGAAG	TCCTTGGAGGCCATGTGGGCCAT
IL-1β	GAAATGCCACCTTTTGACAGTG	TGGATGCTCTCATCAGGACAG
IL-6	TAGTCCTTCCTACCCCAATTTCC	TTGGTCCTTAGCCACTCCTTC
IL-10	GCTCTTACTGACTGGCATGAG	CGCAGCTCTAGGAGCATGTG
IFN-γ	ATGAACGCTACACACTGCATC	CCATCCTTTTGCCAGTTCCTC
HSV-gB	AACGCGACGCACATCAAG	CTGGTACGCGATCAGAAAGC
HSV-gD	GGAGCTGTCCGAGGAATTCAACGCCAC	CATGTTGTTCGGGGTCTCGAGGGGATGGTAAGGCG
β-actin	GGCTGTATTCCCCTCCATCG	CCAGTTGGTAACAATGCCATGT

### 2.8 Apoptosis analysis and cell vitality assay

Apoptosis was assessed using the Apoptosis Detection Kit (KGA1109–20, KeyGEN BioTECH, China). Cells from different treatment groups were collected and resuspended in binding buffer. Annexin V- Alexa Fluor^®^ 647 and propidium iodide (PI) were added to the cell suspension, followed by a 15-minute incubation at room temperature (RT) in the dark. Apoptotic cells were quantified by flow cytometry. Cell vitality was measured using a Cell Counting Kit-8 (CCK8, C0005, TargetMol, China). Cells subjected to different treatments were seeded into 96-well plates and incubated with 100 µl of culture medium containing 10 µl of CCK-8 solution for 4 hours. Absorbance values were recorded at 450 nm using an MRX II microplate reader (Dynex, Chantilly, VA, United States).

### 2.9 Immunofluorescence (IF) analysis

For immunofluorescence, cells were fixed with 4% paraformaldehyde for 30min, treated by 0.5% Triton X-100 (P0096, Beyotime, China) for 15min and blocked by 5% BSA (ST023, Beyotime, China) for 1h. The cells were then incubated with primary antibodies overnight at 4 °C followed by incubation with corresponding secondary antibodies for 2 hours at RT. Nuclei were stained with DAPI by mounting the slides with a DAPI-containing mounting medium (ab104139, Abcam, UK). The antibodies used include: anti-NLRP12 (1:200, YT7568, Immunoway, USA), anti-HSV-gD (1:200, sc-21719, Santa Cruz, USA), Alexa Flour^®^ 488 secondary antibody (1:500, SA00003–2, Proteintech, China), Donkey Anti-Mouse IgG H&L (Alexa Fluor^®^ 647) (1:500, ab150107, Abcam, UK), PE-conjugated anti-MHC II antibody (1:200, 12–5321-82, eBioscience, USA), and Alexa Fluor^®^ 647 Anti-TMS1/ASC antibody (1:200, ab300732, Abcam, UK). The corneas of mice were excised, fixed with 4% paraformaldehyde and incubated in 20mM EDTA for 30min at RT. The corneas were then treated with 0.5% Triton-X 100 for 30min at RT and incubated in 5% donkey serum (SL050, Solarbio, Beijing, China) for 1h at RT. Subsequently, the corneas were incubated overnight at 4°C with antibodies against Alexa Fluor^®^ 488-conjugated anti-F4/80 (1:200, 123120, Biolegend, USA), PerCP-Cyanine5.5-conjugated anti-MHC II (1:200, 107625, Biolegend, USA) and APC-conjugated anti-CD11c (1:200, 117309, Biolegend, USA). After washing, the corneas were radially cut and flat-mounted using DAPI-containing medium. The IF staining was observed using the Leica Thunder system (Leica, Wetzlar, Germany).

### 2.10 Flow cytometry analysis

Single-cell suspensions from mouse submandibular dLNs were obtained by grinding the tissues and filtering through 70μm cell strainers. Staining was performed using PerCP-Cyanine5.5-conjugated anti-CD11b (45–0112-80, eBioscience, USA), Alexa Fluor^®^ 488-conjugated anti-F4/80 (123120, Biolegend, USA), APC-conjugated anti-CD86 (17–0862-82, eBioscience, USA), APC-conjugated anti-CD11c (117309, Biolegend, USA), PE-conjugated anti-MHC II (12–5321-82, eBioscience, USA), Alexa Fluor^®^ 488-conjugated anti-CD4 (100423, Biolegend, USA) and APC-conjugated anti-CD8 (17–0081-81, eBioscience, USA). Flow cytometry analysis was conducted on a BD Accuri C6+ instrument (BD Biosciences, USA). Data analysis was performed using FlowJo V10.4 (FlowJo, LLC, Ashland, OR).

### 2.11 Statistical analysis

All experiments were conducted a minimum of three times. Animals subjects were randomly assigned to experiment groups. No exclusion criteria were pre-established. Statistical analysis was carried out with GraphPad Prism 10.0 (GraphPad, San Diego, CA, USA). Results are presented as mean ± standard error of the mean (SEM). Statistical differences between two groups were determined using a two-tailed unpaired t-test, and one-way or two-way ANOVA was applied for comparisons involving more than two groups. Normal distribution and homogeneity of variance were assessed using the Kolmogorov-Smirnov and Levene’s test, respectively. A *p*-value < 0.05 denoted statistical significance.

## 3 Results

### 3.1 NOD-like receptors participate in the immune response of macrophage following HSV-1 infection

**Figure 1:**
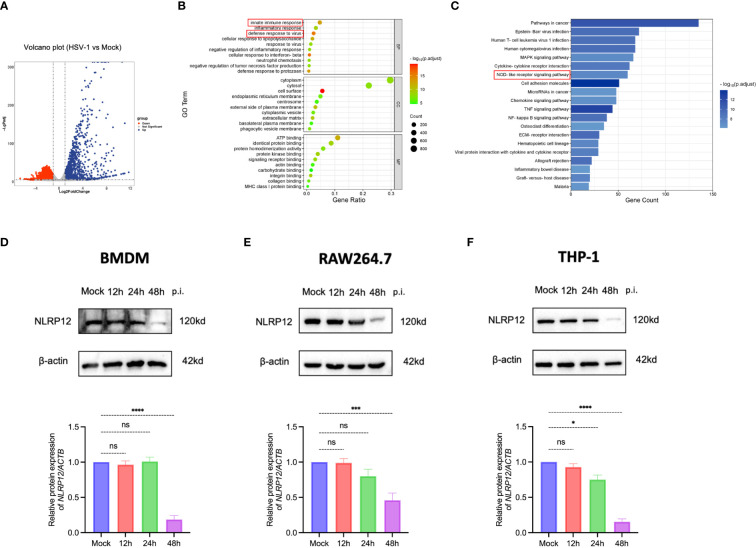
Critical role of NLR signaling pathway in macrophage immune response against HSV-1. **(A)** Volcano plot illustrating the differential expression of 3411 genes, with 1588 upregulated and 1823 were downregulated. A fold change >2 or <0.5 was deemed statistically significant (n=3 per group). **(B)** Gene Ontology (GO) enrichment analysis comparing HSV-1-infected to Mock-infected groups (n=3 per group). **(C)** Kyoto Encyclopedia of Genes and Genomes (KEGG) pathway enrichment analysis comparing HSV-1-infected to Mock-infected groups (n=3 per group). **(D-F)** NLRP12 protein expression levels were evaluated by Western blot in bone marrow-derived macrophages (BMDMs), THP-1 cells, and RAW264.7 cells after mock infection and at 12h, 24h, and 48h post-infection. Western blot bands were normalized to β-actin and set against mock-infected as controls (n=3 per group). All of the data are representative of at least three independent experiments. Data are presented as the mean ± SEM. Statistical differences were determined using one-way ANOVA **(D-F)**. ns, not significnat, *P <0.05, ***P <0.001, ****P <0.0001.

To uncover the immune response triggered by HSV-1 infection in macrophages, we conducted RNA-sequencing analysis of mouse bone marrow-derived macrophages (BMDMs) from both mock-infected and HSV-1-infected groups at 24h post-infection (**Figure 1A**). Gene Ontology (GO) analysis indicated that biological processes related to innate immune response and defense response to virus were significantly altered (**Figure 1B**). Furthermore, Kyoto Encyclopedia of Genes and Genomes (KEGG) analysis identified the NOD-like receptor signaling pathway as one of the most significantly enriched pathways, suggesting a pivotal role for the NOD-like receptors in the defense of BMDMs against HSV-1 infection. We further investigated the expression of NLRP12 in BMDMs, RAW264.7, and THP-1 cells post HSV-1 infection. Western blot results (**Figures 1D-F**) and qRT-PCR results (**supplementary Figure S3A-C**) revealed that NLRP12 expression was notably reduced in all three macrophage cell lines post-infection, indicating that NLRP12 expression is regulated upon HSV-1 infection and likely plays a role in the immune response of macrophages following HSV-1 infection.

### 3.2 Overexpression of NLRP12 enhances viral clearance of macrophage against HSV-1

**Figure 2:**
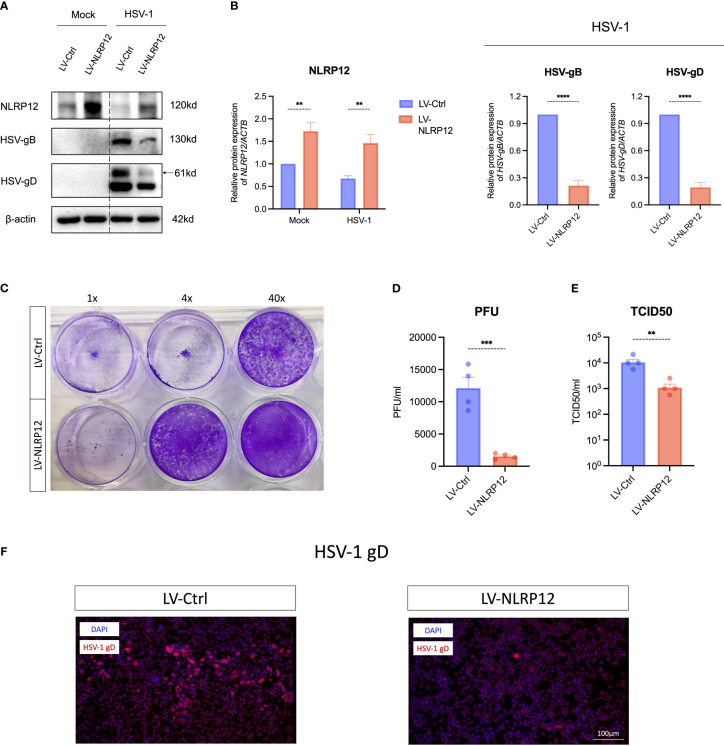
Overexpression of NLRP12 significantly reduces HSV-1 viral load in macrophages. **(A)** Macrophages were transfected with control lentivirus (LV-Ctrl) and lentivirus expressing NLRP12 (LV-NLRP12) were infected with either mock or HSV-1, and protein levels of NLRP12, HSV-gB and HSV-gD were assessed at 24h post-infection. **(B)** Western blot bands were normalized to β-actin and compared against controls (n=3 per group). **(C)** A representative result and **(D)** statistical analysis of plaque assays measuring viral titers in cell culture supernatants at 24h post-infection (n=4 per group). **(E)** TCID50 assay results comparing viral titers in cell culture supernatants between groups at 24h post-infection (n=4 per group). **(F)** Representative immunofluorescence images highlighting HSV-gD in infected cells. All of the data are representative of at least three independent experiments. Data are presented as the mean ± SEM. Statistical differences were determined using two-way ANOVA **(B)** and unpaired t-test **(B, D, E)**. **P< 0.01, ***P < 0.001, ****P < 0.0001.

Lentiviral transfection was employed to overexpress NLRP12 in RAW264.7 macrophages (**Figures 2A, B**). Western blot analysis at 24h post HSV-1 infection revealed significantly lower levels of HSV glycoproteins B (HSV-gB) and D (HSV-gD) in the LV-NLRP12 group compared to the LV-Ctrl group, suggesting that overexpression of NLRP12 strengthened viral clearance in macrophages. Plaque assays (**Figures 2C, D**) and TCID50 measurements (**Figure 2E**) of cell culture supernatants also confirmed the reduced levels of HSV-1 in LV-NLRP12 macrophages. Immunofluorescence staining for HSV-gD (**Figure 2F**) further verified the reduced levels of HSV-1 in the LV-NLRP12 group, suggesting that NLRP12 enhances the antiviral functions of macrophages and improves viral clearance of HSV-1.

### 3.3 NLRP12 enhances macrophage antiviral functions through pyroptosis and downstream signaling

**Figure 3:**
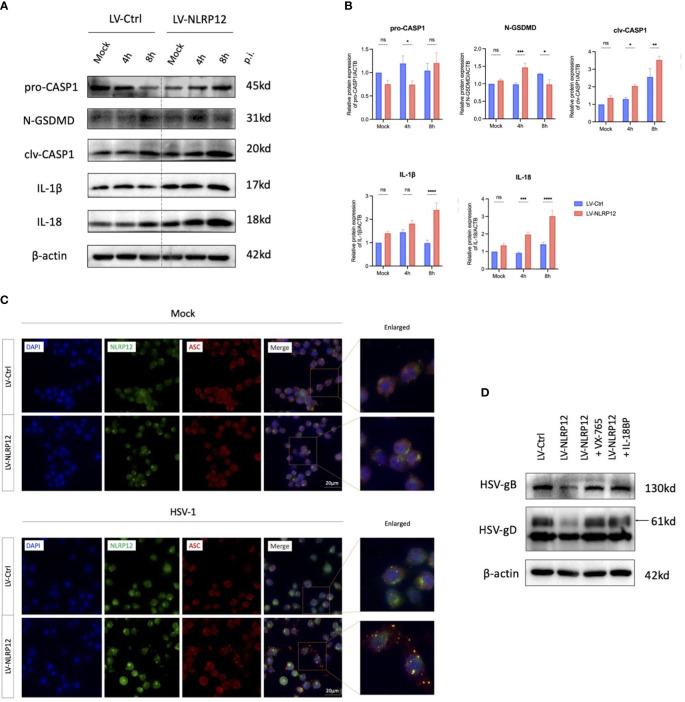
NLRP12 mediates pyroptosis and promotes IL-18 following HSV-1 infection. LV-Ctrl and LV-NLRP12 macrophages were either mock or HSV-1 infected and assessed at specified time points. **(A)** Proteins within the pyroptosis pathway were detected after mock infection and 4h and 8h post-infection. **(B)** Western blot bands were normalized to β-actin and compared against controls (n=3 per group). **(C)** Immunofluorescence images showing NLRP12 (green), ASC (red) and their co-localization (yellow) post mock infection and at 8h post-infection. **(D)** LV-NLRP12 cells were treated with caspase-1 inhibitor VX-765 to inhibit pyroptosis and IL-18 binding protein (IL-18BP) to block active IL-18. Protein levels of HSV-gB and HSV-gD were measured at 8h post-infection. All of the data are representative of at least three independent experiments. Data are presented as the mean ± SEM. Statistical differences were determined using two-way ANOVA **(B)**. ns, not significnat, *P <0.05, **P <0.01, ***P <0.001, ****P <0.0001.

To investigate the mechanisms by which NLRP12 enhances the antiviral functions of macrophages, we initially conducted a series of protein detections of the cell pyroptosis pathway (**Figures 3A, B**). Post-HSV-1 infection, macrophages in the LV-NLRP12 group exhibited earlier and more robust cell pyroptosis. At 4h post-infection, LV-NLRP12 cells showed elevated CASP1 and GSDMD cleavage, followed by higher levels of active IL−1β and IL-18. Immunofluorescence staining of cells either mock-infected or HSV-1-infected at 8h (**Figure 3C**) suggested that, in the mock condition, NLRP12 and ASC were distributed in the cytoplasm without colocalization. Post HSV-1 infection, the LV-NLRP12 group displayed more specks and a higher level of colocalization, indicating increased formation of inflammasomes and a higher degree of cell pyroptosis. Furthermore, the use of the caspase-1 inhibitor VX-765 or the IL-18 antagonist IL-18BP in LV-NLRP12 macrophages reduced the enhanced antiviral function by inhibiting Caspase-1 activity or the key effector molecule IL-18 (**Figure 3D**). This indicated the important role of pyroptosis-induced IL-18 in the enhanced antiviral response.

**Figure 4:**
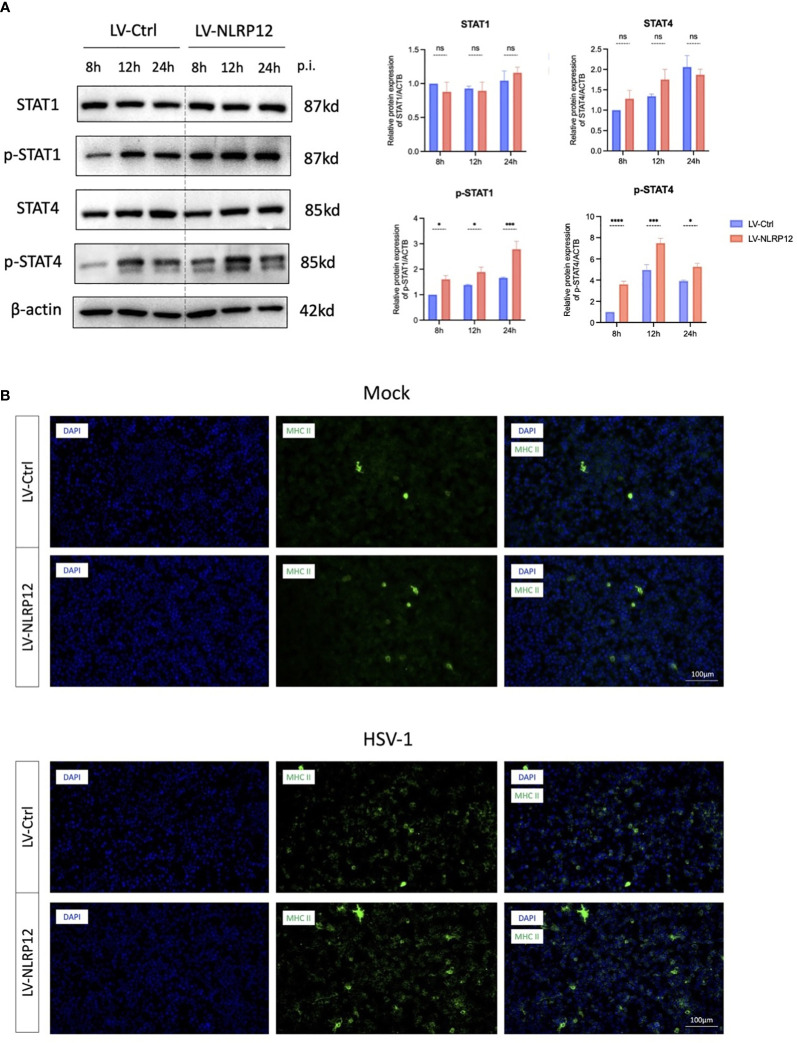
NLRP12 enhances antiviral functions through IL-18 downstream JAK-STAT signaling. **(A)** Protein levels of STAT1, p-STAT1, STAT4, pSTAT4 were assessed at 8h, 12h and 24h post-infection in both groups. Western blot bands were normalized to β-actin and compared against controls (n=3 per group). **(B)** Immunofluorescence images demonstrating MHC II^+^ cell staining. All of the data are representative of at least three independent experiments. Data are presented as the mean ± SEM. Statistical differences were determined using two-way ANOVA **(A)**. ns, not significnat, *P <0.05, ***P <0.001, ****P <0.0001.

Consequently, we focused on IL-18, the important downstream molecule of cell pyroptosis to further explore the link between NLRP12-mediated cell pyroptosis and subsequent antiviral immune response. IL-18, also known as IFN-γ inducing factor, has been reported to stimulate the production and release of IFN−γ via the JAK/STAT4 signaling pathway (Wu et al., 2019). IFN−γ, in turn, can exert antiviral immune functions through the JAK/STAT1 signaling pathway, including upregulating the production of MHC II (Thelemann et al., 2014; Liu et al., 2020). Thus, we detected the activation of STAT1 and STAT4 at 8, 12, and 24 hours post-infection as possible downstream signaling of the induced pyroptosis. Western blot analysis (**Figure 4A**) indicated the presence of elevated phosphorylated-STAT4 (pSTAT4) and phosphorylated-STAT1 (pSTAT1) levels at 8, 12, and 24 hours post-infection. These findings suggest that HSV-1 infection triggered an early pyroptotic response in LV-NLRP12 macrophages, and the resultant release of IL-18 could induce antiviral immune cascade in neighboring cells, ultimately enhancing the overall clearance of HSV-1.

Additionally, immunofluorescence staining showed that LV-NLRP12 macrophages expressed higher levels of MHC II post-infection (**Figure 4B**), indicating enhanced antigen-presenting functions and antiviral immune responses. The expression levels of MHC II were low in both groups under mock-infection. However, the LV-NLRP12 group showed an increase in MHC II^+^ cells post-infection, as well as signs of heightened polarization characterized by an increase in cell size and the presence of more dendrites and extensions. This indicated immunomodulatory role of LV-NLRP12 macrophages requires further validation in the *in vivo* environment.

### 3.4 Overexpression of NLRP12 protects cell vitality and balances macrophage functions

**Figure 5:**
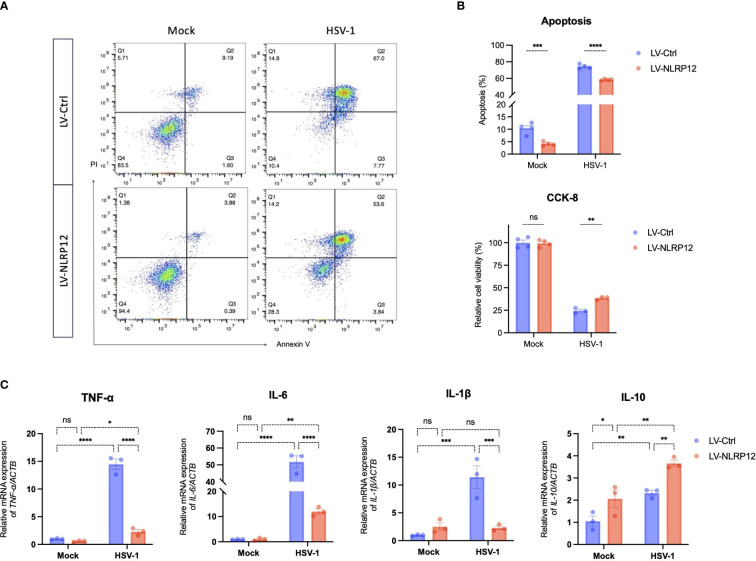
Overexpression of NLRP12 attenuates virus-induced cytotoxicity and inflammation in macrophages. LV-Ctrl and LV-NLRP12 macrophages were either mock or HSV-1 infected and analyzed at 48h post-infection. **(A)** Apoptosis rates were examined by flow cytometry using Annexin-V - Alexa Fluor^®^ 647/PI staining. **(B)** Quantification analysis of Annexin-V^+^/PI^+^ apoptosis rates (double Annexin-V + PI positive) and cell vitality using CCK-8 assay across different groups (n=4 per group). **(C)** qRT-PCR analysis measuring expression levels of TNF-α, IL-6, IL-1β, and IL-10 in different groups (n=3 per group). All of the data are representative of at least three independent experiments. Data are presented as the mean ± SEM. Statistical differences were determined using two-way ANOVA **(B, C)**. ns, not significnat, *P <0.05, **P <0.01, ***P <0.001, ****P <0.0001.

The above results showed that overexpression of NLRP12 enhanced antiviral functions and significantly reduced the viral load at 24h post-infection. We further performed cell apoptosis analysis and CCK-8 test to assess the effects of NLRP12 overexpression at later post-infection stage. At 48h post-infection, analysis of the cell apoptosis rate (**Figures 5A, B**) showed a significant decrease in the LV-NLRP12 group, and cell vitality assay using CCK-8 test (**Figure 5B**) indicated improved cell vitality in the LV-NLRP12 group compared to the control group. These results suggest that NLRP12 overexpression could diminish virus-induced cytotoxicity at later post-infection stage. We also examined the mRNA expression of cytokines in both groups at 48h post-infection (**Figure 5C**). The levels of pro-inflammatory cytokines TNF-α, IL-6 and IL-1β were significantly reduced by 3–5-fold in the LV-NLRP12 group compared to the control group, whereas the level of anti-inflammatory cytokine IL-10 was significantly elevated by 2-fold. These findings suggest that after rapid clearance of the virus, NLRP12 could attenuate virus-induced inflammatory response and potentially balance macrophage functions to favor the repair of infected tissues.

### 3.5 Adoptive transfer of NLRP12-overexpressing macrophages enhances antiviral immune response in herpes simplex keratitis mice

**Figure 6:**
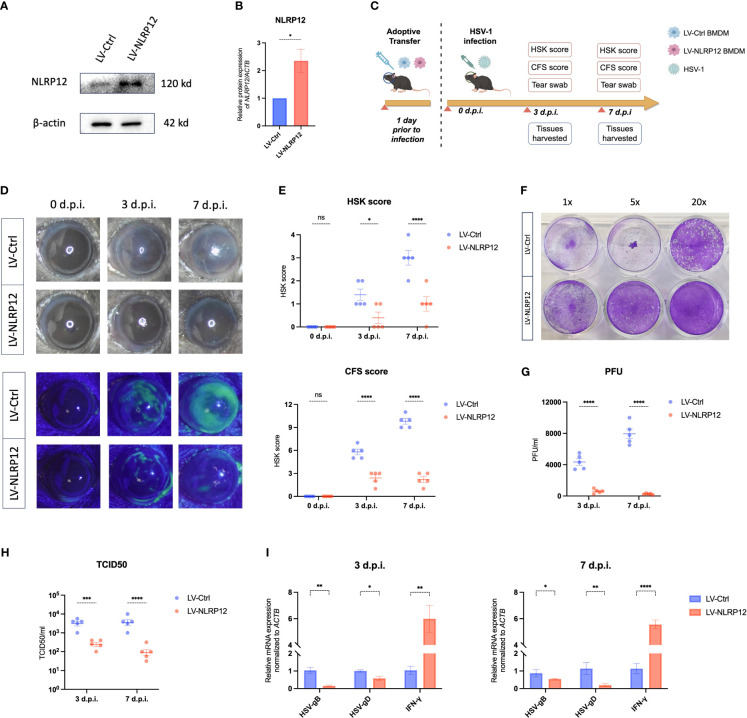
Adoptive transfer of macrophages overexpressing NLRP12 alleviates HSK and reduces viral load. **(A)** BMDMs were transfected with LV-Ctrl or LV-NLRP12. Protein levels of NLRP12 were detected and **(B)** western blot bands were normalized to β-actin and compared against controls (n=3 per group). **(C)** Diagram of adoptive transfer procedure and HSK animal model timeline. **(D)** Representative images of corneal lesions in HSK mice from LV-Ctrl and LV-NLRP12 groups, observed under natural light (top) and under cobalt blue lights after fluorescein sodium staining (bottom). **(E)** Clinical assessment of HSK scores and corneal fluorescein staining (CFS) scores (n=5 per group). **(F)** Representative result and **(G)** statistical analysis of viral titers from plaque assays of tear swabs at 3 and 7 days post-infection (n=5 per group). **(H)** TCID50 assay results of tear swabs at 3 and 7 days post-infection (n=5 per group). **(I)** qRT-PCR analysis detecting expression of HSV-1 gB, HSV-1 gD, and IFN-γ in corneas at 3 and 7 days post-infection (n=5 per group). All of the data are representative of at least three independent experiments. Data are presented as the mean ± SEM. Statistical differences were determined using two-way ANOVA **(E, G–I)**. ns, not significnat, *P <0.05, **P <0.01, ***P <0.001, ****P <0.0001.

To corroborate the *in vivo* role of NLRP12-overexpressing macrophages, we conducted ocular adoptive transfer of NLRP12-overexpressing BMDMs. Lentiviral transfection was employed to induce NLRP12 overexpression in BMDMs, as demonstrated in **Figures 6A, B**. According to the schematic timeline (**Figure 6C**), mice received subconjunctival injections of LV-Ctrl or LV-NLRP12 BMDMs one day before corneal HSV-1 inoculation. Specified evaluations were conducted at designated post-infection intervals. Results in **Figures 6D, E** showed that adoptive transfer of LV-NLRP12 BMDMs alleviated HSK clinical symptoms. The corneas of HSK mice treated with LV-NLRP12 BMDMs maintained transparency and exhibited fewer instances of cloudiness and neovascularization. Fluorescein sodium staining demonstrated less corneal epithelial damage in the LV-NLRP12 group, with notable recovery by the seventh day post-infection. Plaque assay and TCID50 results from tear swabs indicated a significant reduction of HSV-1 viral load in the tears of mice from the LV-NLRP12 group compared to the LV-Ctrl group (**Figures 6F–H**). Additionally, levels of HSV-gB and HSV-gD in corneas from the LV-NLRP12 group were markedly decreased (**Figure 6I**), indicating effective viral clearance through adoptive transfer of LV-NLRP12 BMDMs. Concurrently, the levels of IFN−γ in the corneas of the LV-NLRP12 group showed a significant increase, suggesting an improved antiviral immune status.

**Figure 7:**
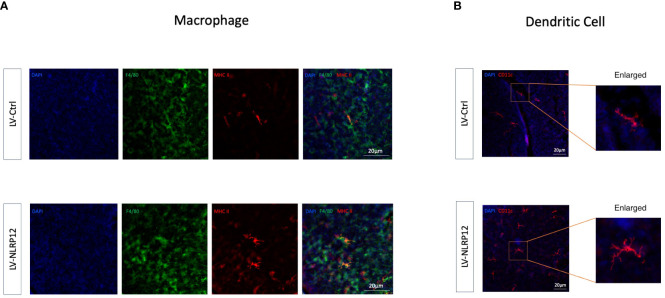
Macrophages overexpressing NLRP12 improves immune response in the corneas of HSK mice. **(A)** Immunofluorescence staining images of whole-mount mouse corneas at 3 days post-infection showing F4/80 (green), MHC II (red) and their co-localization (yellow). **(B)** Immunofluorescence staining images of whole-mount mouse corneas at 3 days post-infection highlighting CD11c. All of the data are representative of at least three independent experiments.

**Figure 8:**
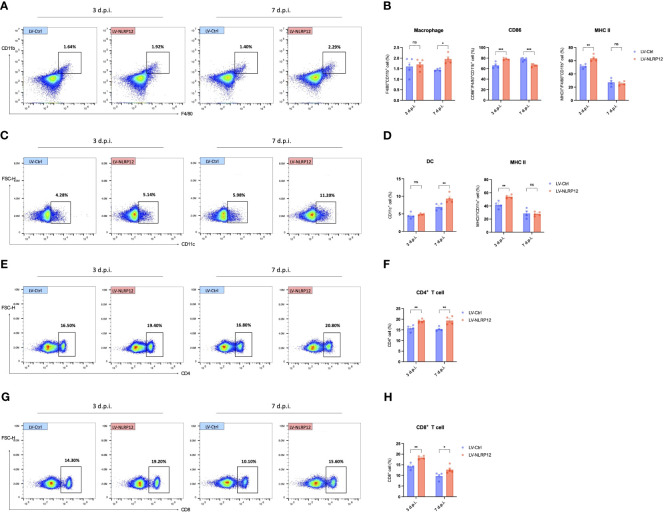
Macrophages overexpressing NLRP12 promotes a comprehensive immune response in the dLNs during HSK. **(A)** Representative flow cytometry plots of CD11b^+^ cells in the dLNs at 3 and 7 days post-infection. **(B)** Bar charts indicating the percentage of CD11b^+^cells in the dLNs, CD86^+^/CD11b^+^ cells and MHC II^+^/CD11b^+^ cells (n=5 per group). **(C)** Representative flow cytometry plots of CD11c^+^ cells in the dLNs at 3 and 7 days post-infection. **(D)** Bar charts indicating the percentage of CD11c^+^cells in the dLNs and MHC II^+^/CD11b^+^ cells (n=5 per group). **(E)** Representative flow cytometry plots of CD4^+^ cells and **(G)** CD8^+^ cells in the dLNs at 3 and 7 days post-infection. **(F)** Bar charts representing the percentage of CD4^+^ cells and CD8^+^ cells H) in the dLNs (n=5 per group). Gating strategy of flow cytometry analysis of dLNs is shown in **Supplementary Figure 4**. All of the data are representative of at least three independent experiments. Data are presented as the mean ± SEM. Statistical differences were determined using two-way ANOVA **(B, D, F, H)**. ns, not significnat, *P <0.05, **P <0.01, ***P <0.001.

Further analysis using immunofluorescent staining of whole-mount mouse corneas revealed that corneal-infiltrating macrophages expressed heightened levels of MHC II and exhibited increased branching in the LV-NLRP12 group (**Figure 7A**). Dendritic cells (DCs) infiltrating the corneas similarly manifested greater maturity in the LV-NLRP12 group, as evidenced by augmented size, branching complexity, and dendritic tips (**Figure 7B**), which likely aligned with the elevated IFN-γ levels within the corneas of these mice. The immunological responses in the submandibular draining lymph nodes were further examined using flow cytometry analysis. On the third day post-infection, while the number of macrophages in the dLNs did not differ significantly, those from the LV-NLRP12 group had a higher proportion of CD86^+^ and MHC II^+^ cells (**Figures 8A, B**). On the seventh day post-infection, the LV-NLRP12 group sustained an increased macrophage count in the dLNs but with a reduced percentage of CD86 positivity, indicating a potential shift towards promoting corneal repair functions. Analysis of DCs revealed that on the third day post-infection, DC counts in the dLNs did not differ significantly, but those from the LV-NLRP12 group exhibited a higher MHC II positivity proportion (**Figures 8C, D**). This was consistent with the above findings of macrophages, indicating an enhanced antigen-presenting response in both cell types. On the seventh day post-infection, the LV-NLRP12 group also maintained a higher DC count. Analysis of adaptive immune cells in the dLNs revealed that the LV-NLRP12 group exhibited a significantly higher count of both CD4^+^ T cells (**Figures 8E, F**) and CD8^+^ T cells (**Figures 8G, H**) on the third and seventh days post-infection. These findings suggest that corneal adoptive transfer of LV-NLRP12 BMDMs also regulated the antiviral immune response in the draining lymph nodes through a cascade of immunological reactions, effectively enhancing the clearance of HSV-1 *in vivo*.

## 4 Discussion

**Figure 9:**
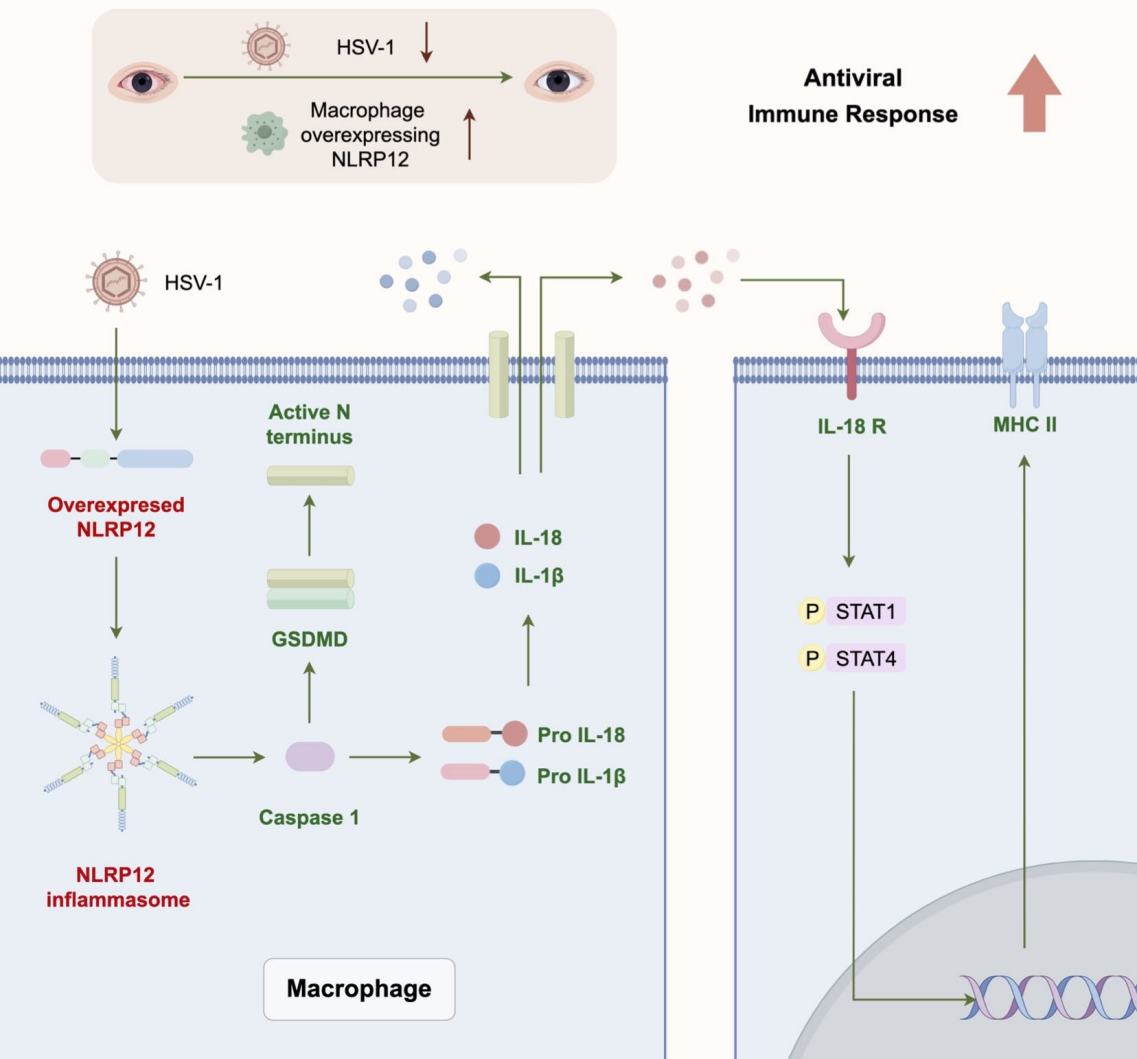
Graphic Abstract. NLRP12 modulates the antiviral immune response of macrophages through pyroptosis and downstream signaling, and enhances the subsequent immune response cascade.

This study provides novel insights into the immunomodulatory role of NLRP12 against HSV-1 infection. The results from *in vitro* and *in vivo* experiments demonstrate that NLRP12 modulated the antiviral functions of macrophages and the subsequent immune response cascade through pyroptosis and downstream signaling (**Figure 9**).

RNA-sequencing analysis of BMDMs has established the innate immune response and defense against the virus as significantly altered biological processes upon HSV-1 infection. This supports the established role of macrophages as first responders of the innate immune system to pathogen invasion (Mu et al., 2021). The significant alteration in the NOD-like receptor signaling pathway further emphasizes the importance of these receptors in recognizing pathogens and initiating immune response. Our observation of decreased NLRP12 expression in various macrophage cell lines post-HSV-1 infection prompts questions about potential immune evasion strategies employed by HSV-1 (Greenan et al., 2021). It is plausible that HSV-1 may actively suppress NLRP12 to dampen the immune response, as the restoration of NLRP12 by overexpression appears to counteract this effect and promote viral elimination. This reflects similar strategies of HSV-1 to evade other inflammasomes, such as inhibiting the AIM2 inflammasome via the VP22 protein (Maruzuru et al., 2018) and disrupting NLRP3 and IFI16 inflammasome activation through ICP0 and proteasome pathways (Johnson et al., 2013). The inverse correlation between NLRP12 expression and HSV-1 replication necessitates further investigation to understand this dynamic and its implications.

The overexpression of NLRP12 in RAW264.7 macrophages led to a significant reduction in viral loads, suggesting that NLRP12 maybe a crucial link between innate detection and the enhancement of immune functions (Tuladhar and Kanneganti, 2020). NLRP12’s role in promoting cell pyroptosis and subsequent IL-18 production highlights a distinct immunological pathway. The finding that antiviral functions were compromised when pyroptosis or IL-18 signaling was inhibited underscores the significance of these processes in the NLRP12-mediated antiviral response. It is noteworthy that antiviral immune responses engage not only the NLR signaling pathway but also various other pathways, including the RIG-I-like receptor (RLR) pathway, the Toll-like receptor (TLR) pathway, and the cGAS-STING pathway (Seth et al., 2006). These pathways interact closely, potentially enhancing or modulating the NLRP12-mediated immune response to viruses. NLRP12 may also protect against HSV-1 infection through mechanisms beyond pyroptosis. NLRP12 has been reported to regulate the function of TRIM25 via RIG-I K63-linked ubiquitylation (Chen et al., 2019). NLRP12 also facilitates type I IFNs production by interacting with heat shock protein 90 (HSP90) and inhibit dengue virus replication (Li et al., 2021). Further research is needed to explore the potential signaling events both upstream and downstream of NLRP12 activation and the interplay of NLRP12 with other critical antiviral pathways.

Unlike most other NLRs, the regulatory role of NLRP12 appears to be context-dependent and may involve both inflammasome-dependent and inflammasome-independent pathways (Tuladhar and Kanneganti, 2020). This study augments the growing body of research investigating the role of NLRP12 inflammasome in infectious diseases (Vladimer et al., 2012; Ataide et al., 2014; Zaki et al., 2014; Znalesniak et al., 2017; Rajabi et al., 2022). Additionally, NLRP12 has also been shown to downregulate both the canonical and non-canonical NF-κB pathways by impeding downstream signaling of TLRs and diminishing ERK activation (Zamoshnikova et al., 2016; Chang et al., 2017). The full scope of NLRP12’s regulatory abilities in maintaining immune homeostasis remains further clarification.

Moreover, the antiviral functions and mechanisms of macrophages are multifaceted (Yu et al., 2022), involving various processes and pathways essential for HSV-1 defense that warrant additional exploration. Beyond its expression in macrophages, NLRP12 is associated with dendritic cells (Valadares et al., 2023) and neutrophils (Ulland et al., 2016; Hornick et al., 2018; Wang et al., 2018), which also participate in combating HSV-1 infection and HSK. Further research is required to elucidate the significant roles of NLRP12 in other innate immune cells and to confirm the *in vitro* findings within the intricate *in vivo* environment.

*In vivo*, the adoptive transfer of NLRP12-overexpressing BMDMs reduced HSK clinical symptoms and HSV-1 viral load, which is a strong support of the therapeutic potential of manipulating NLRP12 expression in macrophages. The elevated IFN-γ levels and increased antigen presentation capacity of infiltrating macrophages and DCs is indicative of a more effective antiviral immune environment in the cornea. The altered dynamics in the dLNs with increased numbers of macrophages and DCs as well as CD4^+^ and CD8^+^ T cells also align with the established role of the dLNs as sites for orchestrating systemic immune response (He et al., 2022; Shen et al., 2024). The enhancement of these immune cells suggests improved antigen presentation and T cell activation, which leads to a robust and efficient adaptive immune response to clear the virus (Zhu and Viejo-Borbolla, 2021). Future investigations should aim to validate these findings in human cells and other animal models, and extend the findings to encompass all strains of HSV-1 and other herpesviruses.

The present study has several limitations that should also be considered when interpreting the results. Firstly, the fluorescence channels of the FACS instrument restricted the number of markers that could be analyzed simultaneously. Future analyses of the immune cells would benefit from the inclusion of a viability stain and additional indicators. Secondly, only female mice were used in this study, and the sex of the animals may influence the study results. Therefore, the findings should be verified in studies involving both male and female mice.

Overall, our findings demonstrate the role of NLRP12 in mediating the immune response of macrophages to HSV-1, with implications for *in vivo* management of HSK. These findings open up avenues for the development of therapeutic strategies to capitalize on the antiviral and immunomodulatory properties of NLRP12.

## Data availability statement

The RNA-seq data presented in the study are deposited in the Sequence Read Archive (SRA) repository, accession number PRJNA1135544. The original contributions presented in the study are included in the article/**Supplementary Material**, further inquiries can be directed to the corresponding author.

## Ethics statement

The animal study was approved by The Animal Research Ethics Committee of Nanjing Drum Tower Hospital. The study was conducted in accordance with the local legislation and institutional requirements.

## Author contributions

JJ: Conceptualization, Data curation, Formal analysis, Methodology, Project administration, Writing – original draft. DZ: Data curation, Formal analysis, Methodology, Validation, Writing – review & editing. WL: Data curation, Formal analysis, Investigation, Visualization, Writing – review & editing. JY: Data curation, Investigation, Writing – review & editing. FY: Methodology, Visualization, Writing – review & editing. JL: Formal analysis, Investigation, Writing – review & editing. KH: Conceptualization, Funding acquisition, Project administration, Resources, Supervision, Writing – review & editing.
