# Supplementary material for: Overexpression of NLRP12 enhances macrophage immune response and alleviates herpes simplex keratitis
Source: Front Cell Infect Microbiol. 2024 Jul 25;14:1416105. doi: 10.3389/fcimb.2024.1416105 (PMC11306119; doi:10.3389/fcimb.2024.1416105)

Supplementary Figure S1: Isolation and identification of BMDMs.

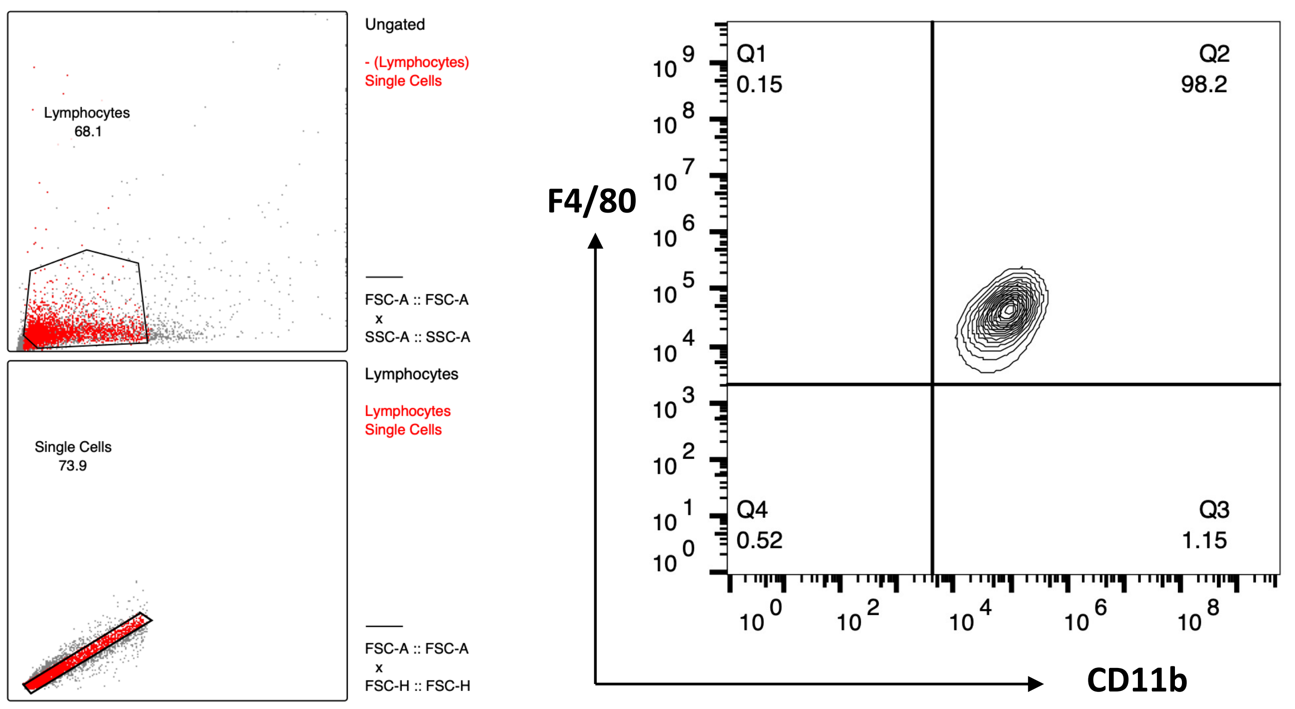

Flow Cytometry analysis of BMDM formation.

BMDMs were isolated and differentiated according to previous established protocols (doi:10.1007/978-1-0716-2128-8_8.).
The cells were first gated on FSC and SSC to remove debris and conjugates. Mature BMDMs were identified as CD11b+F4/80+ populations.

Supplementary Figure S2: Subconjunctival injection and ocular HSV-1 infection.

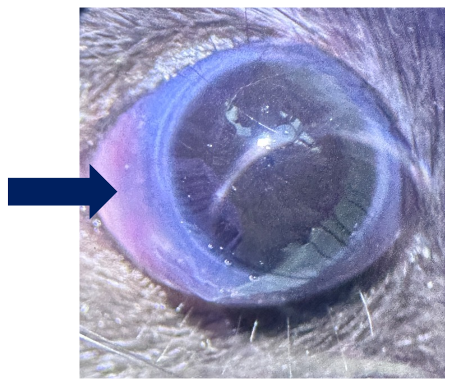

A. Subconjunctival injection.

1. A 33G needle was filled with the sterile solution intended for injection.
2. The eyelid of the anesthetized mouse was gently pulled down to expose the conjunctival sac. The needle was held at a slight angle and inserted into the subconjunctival space, located between the sclera and conjunctiva, near the edge of the eye.
3. Light pressure was applied on the injection site for several minutes to prevent leakage and ensure proper administration of the solution.

(Arrow) A crescent swell of the conjunctival sac can be observed after successful injection.

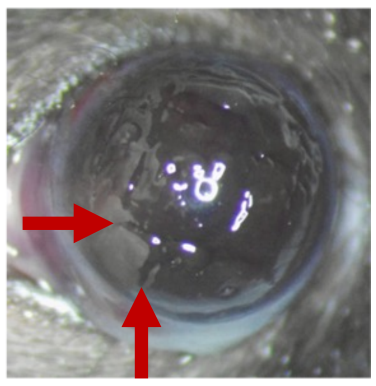

B. Corneal scratching and HSV-1 infection.

1. The eyelid of the anesthetized mouse was gently pulled down to expose the cornea. The tip of a 33G needle was used to scratch the corneal epithelium, making 3-5 horizontal and 3-5 vertical scratches. (indicated by arrows)
2. A volume of 5ul of HSV strain Mckrae (1 × 10^6 PFU/ml) was applied to the freshly scratched cornea.
3. The eyelid was gently massaged to enhance the absorption of the virus into the cornea.

Supplementary Figure S3: supplementary qRT-PCR results.

A

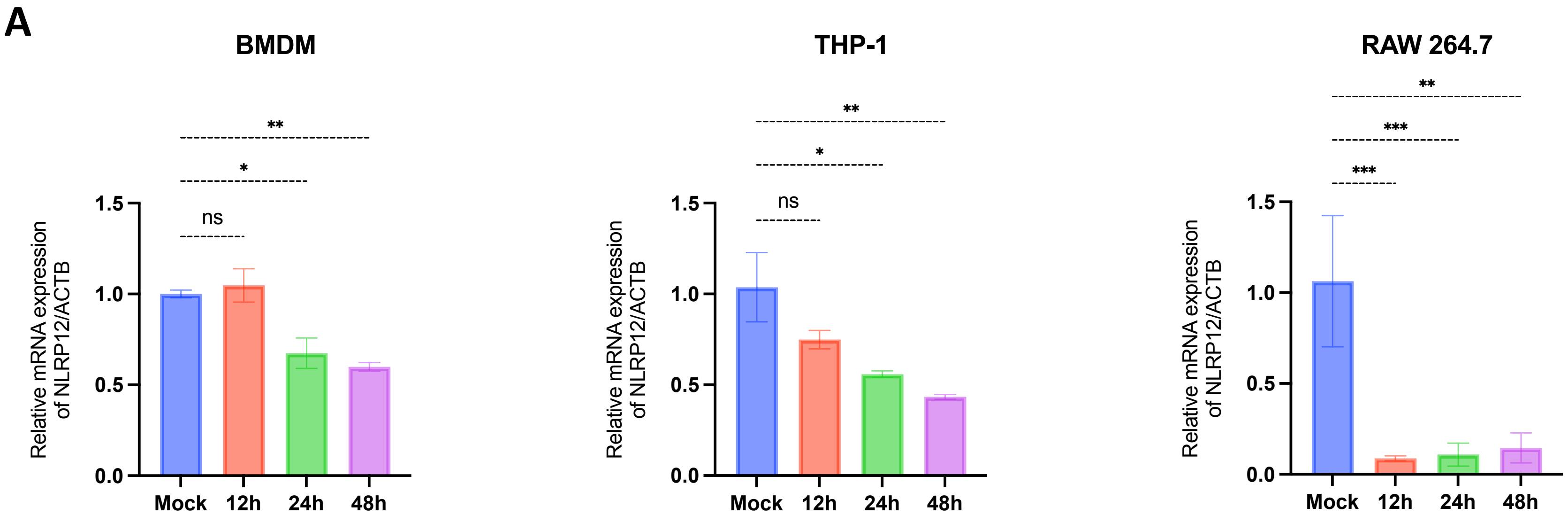

A. NLRP12 mRNA expression in macrophage cell lines.

BMDMs, THP-1 cells and RAW264.7 cells were infected with HSV-1. The mRNA expression of NLRP12 was detected at specified time points using qRT-PCR.

B

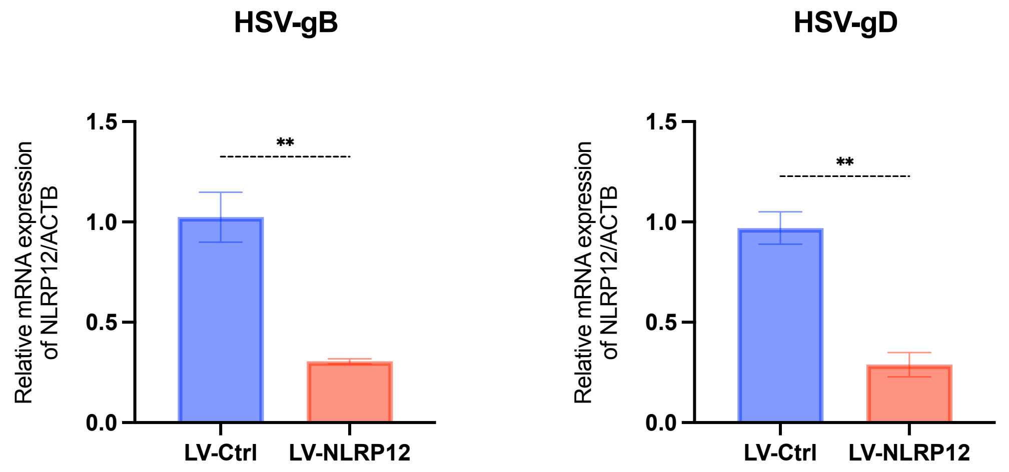

B. HSV gB and gD mRNA expression.

At 24h post-infection, cells from the LV-Ctrl group and LV-NLRP12 group were collected for qRT-PCR analysis. The mRNA expression of HSV-gB and HSV_gD was presented.

Supplementary Figure S4: Gating strategy for flow cytometry analysis of dLNs.

A. The cells in the single cell suspensions of the dLNs is predominantly leukocytes (>98%).

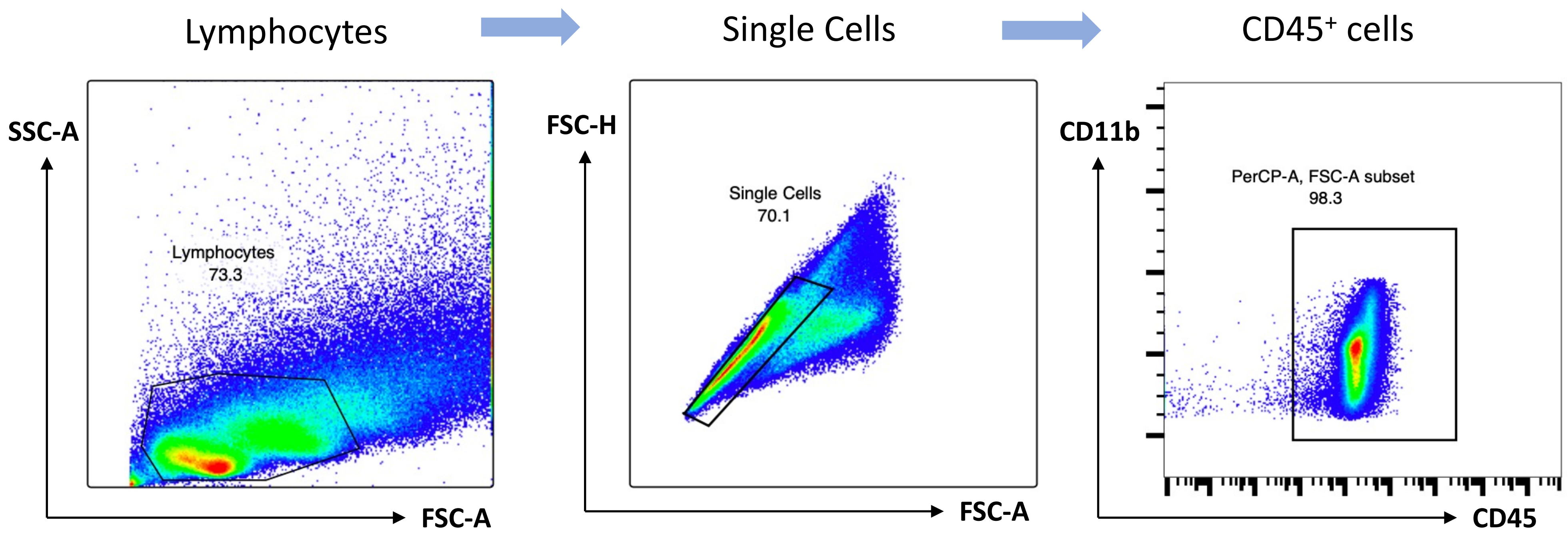

Supplementary Figure S4: Gating strategy for flow cytometry analysis of dLNs.

B. Gating strategy for Macrophages.

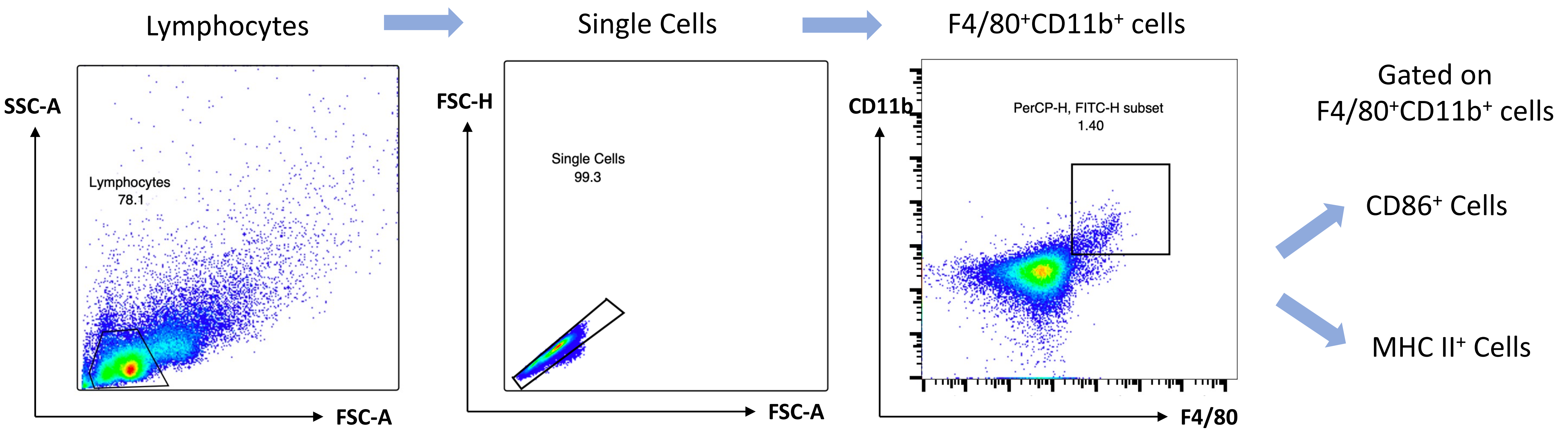

C. Gating strategy for DCs.

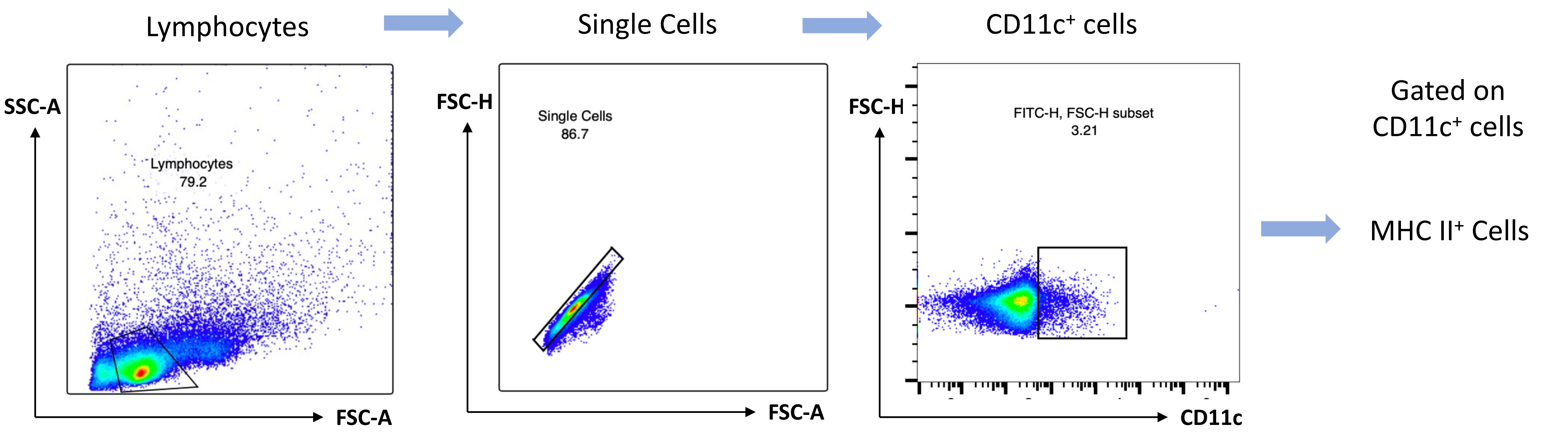

D. Gating strategy for T cells.

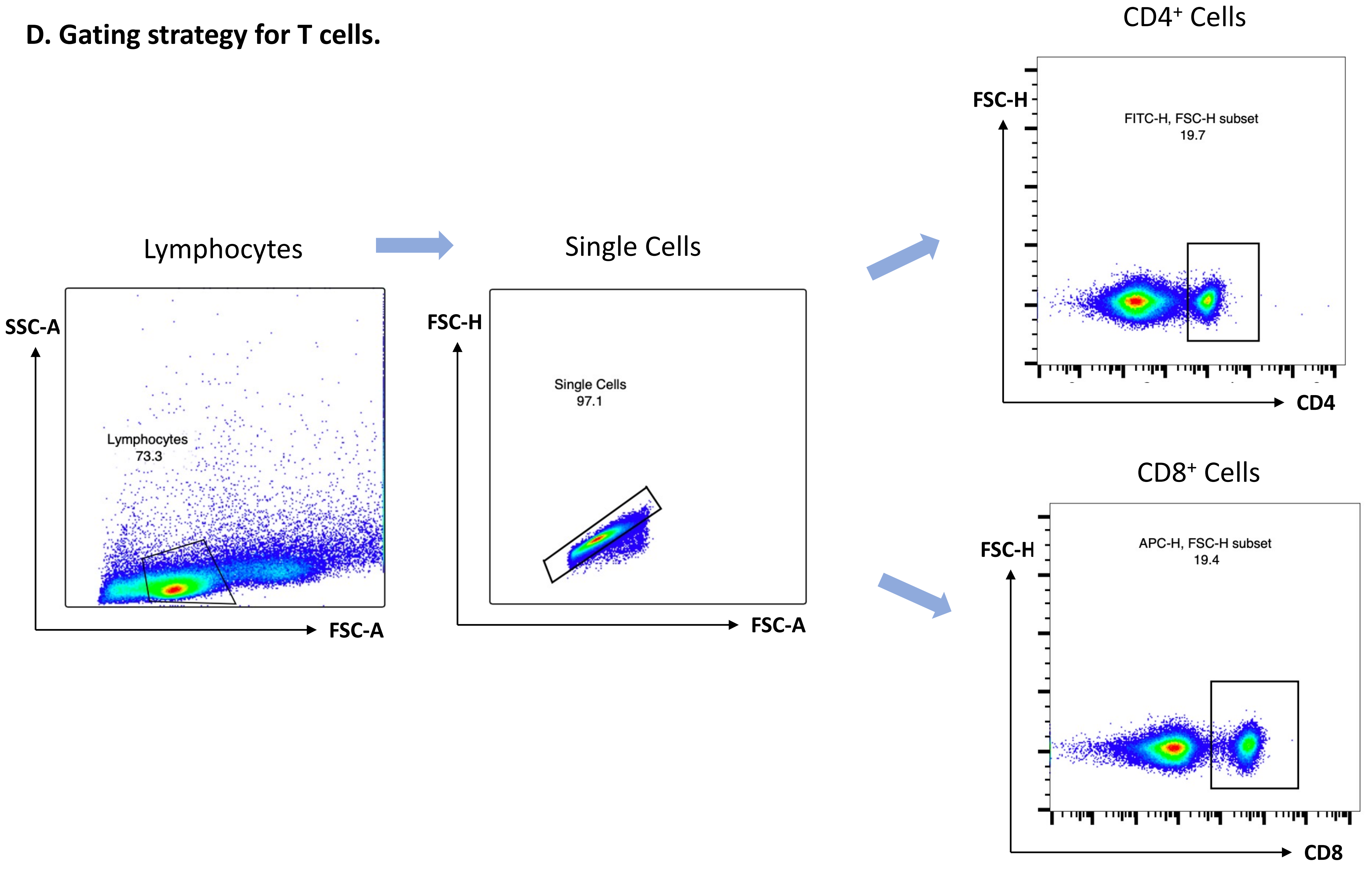

Supplement: Supplementary file 1 [file DataSheet_1.pdf]
